# Disparities in risk perception and low harm reduction services awareness, access, and utilization among young people with newly reported hepatitis C infections in California, 2018

**DOI:** 10.1186/s12889-021-11492-3

**Published:** 2021-07-21

**Authors:** Alison R. Ohringer, David P. Serota, Rachel L. McLean, Lauren J. Stockman, James P. Watt

**Affiliations:** 1 Division of Infectious Diseases, Department of Medicine, University of Miami Miller School of Medicine, Miami, FL USA; 2 Office of Viral Hepatitis Prevention, Sexually Transmitted Diseases Control Branch, Division of Communicable Disease Control, Center for Infectious Diseases, California Department of Public Health, 850 Marina Bay Parkway, Building P, 2nd Floor, Richmond, CA 94804 USA; 3 Division of Communicable Disease Control, Center for Infectious Diseases, California Department of Public Health, Richmond, CA USA

**Keywords:** Hepatitis C, Epidemiology, HCV, Injection drug use, Risk, Screening, Needle and syringe exchange, Harm reduction, Naloxone, Incarceration

## Abstract

**Background:**

Newly reported hepatitis C virus (HCV) infections in California increased 50% among people 15–29 years of age between 2014 and 2016. National estimates suggest this increase was due to the opioid epidemic and associated increases in injection drug use. However, most of California’s 61 local health jurisdictions (LHJs) do not routinely investigate newly reported HCV infections, so these individuals’ risk factors for infection are not well understood. We sought to describe the demographics, risk behaviors, and utilization of harm reduction services in California’s fastest-rising age group of people with newly reported hepatitis C infections to support targeted HCV prevention and treatment strategies.

**Methods:**

California Department of Public Health invited LHJs to participate in enhanced surveillance if they met criteria indicating heightened population risk for HCV infection among people ages 15–29. From June–December 2018, eight LHJs contacted newly reported HCV cases by phone using a structured questionnaire.

**Results:**

Among 472 total HCV cases who met the inclusion criteria, 114 (24%) completed an interview. Twenty-seven percent of respondents (*n* = 31) had ever been incarcerated, of whom 29% received a tattoo/piercing and 39% injected drugs while incarcerated. Among people who injected drugs (PWID)—36% (*n* = 41) of all respondents—68% shared injection equipment and many lacked access to harm reduction services: 37% knew of or ever used a needle exchange and 44% ever needed naloxone during an overdose but did not have it. Heroin was the most frequently reported injected drug (*n* = 30), followed by methamphetamine (*n* = 18). Pre-diagnosis HCV risk perception varied significantly by PWID status and race/ethnicity: 76% of PWID vs. 8% of non-PWID (*p* < 0.001), and 44% of non-Hispanic White respondents vs. 22% of people of color (POC) respondents (*p* = 0.011), reported thinking they were at risk for HCV before diagnosis. Eighty-nine percent of all respondents reported having health insurance, although only two had taken HCV antiviral medications.

**Conclusions:**

Among young people with HCV, we found limited pre-diagnosis HCV risk perception and access to harm reduction services, with racial/ethnic disparities. Interventions to increase harm reduction services awareness, access, and utilization among young PWID, especially young PWID of color, may be warranted.

**Supplementary Information:**

The online version contains supplementary material available at 10.1186/s12889-021-11492-3.

## Background

Nearly 2.4 million people in the United States are estimated to have chronic hepatitis C virus (HCV) infection, with many remaining undiagnosed [1]. In California, newly reported HCV infections increased 50% among people 15–29 years of age between 2014 and 2016 [2]. However, most of California’s 61 local health jurisdictions (LHJs) do not have the capacity to routinely investigate newly reported HCV infections, so these individuals’ risk factors for infection are not well understood. National estimates suggest this increase is due to the opioid epidemic and associated increases in injection drug use (IDU) [3]. One meta-analysis estimated the United States had 2.2 million people who inject drugs (PWID), of whom an estimated 10.5% were less than 25 years of age [4]. Another study estimated U.S. lifetime drug use prevalence to be 2.6% (6.6 million), with a past-year injection drug use prevalence of 0.30%, totaling approximately three-quarter million PWID [5]. The number of PWID in California, however, is not well documented. In addition, data on hepatitis C risk and harm reduction service utilization among young PWID in California are limited to major cities (namely San Francisco) and are not necessarily generalizable to the entire state. Longitudinal analysis of predictors of hepatitis C incidence among PWID under 30 years of age in San Francisco found that housing instability was associated with HCV acquisition, with those unhoused versus housed at baseline having a 1.9-fold increased infection risk [6].

Outside of California, research has found differences in syringe exchange program (SEP) utilization by race and ethnicity, with African American/Black respondents less likely than White respondents to access syringes from SEPs, regardless of distance, and Latino respondents less likely to access syringes from SEPs as distance increased [7]. Potential factors influencing SEP access among African American/Black and Latino respondents included racial profiling, obtaining syringes from secondary sources (such as peers), mistrust of police, and stigma [7]. A separate analysis of SEP utilization before and after a targeted police operation that increased police presence to reduce open drug sales in Philadelphia found that SEP utilization decreased after the operation, with decreased utilization most pronounced among African American/Black and male participants [8]. A study of PWID perceptions of police presence in Baltimore found syringe sharing was independently associated with asking to borrow equipment in neighborhoods with perceived heightened police activity [9].

Modeling studies among people who inject drugs suggest a combination of syringe access, medications for opioid use disorder (MOUD), and HCV treatment is needed to reduce hepatitis C incidence and prevalence [10]. The U.S. Centers for Disease Control and Prevention (CDC) recommends comprehensive prevention programs, including syringe access, MOUD, and hepatitis C testing and treatment to prevent transmission of HCV and other infectious diseases among PWID [9]. CDC has found SEPs to be safe, effective, cost-saving, and to not increase illegal drug use or crime [11]. In California, access to harm reduction services varies greatly between and within counties. Syringe exchange programs can be either authorized by the state health department or city/county governments, or started by a physician without requiring state authorization, but many counties have no SEPs at all [12]. As of 2018, when this survey was conducted, there were more than 40 SEPs operating in California [13]. Access to harm reduction services for opioid use, such as medications for opioid use disorder and naloxone for the reversal of potentially fatal opioid overdoses, is more widespread thanks to statewide and local responses to the opioid epidemic, including a major, multifaceted MOUD expansion project and free naloxone distribution project [14, 15]. However, given the pivotal role SEPs play in distributing naloxone to PWID, naloxone coverage among PWID may be more limited in jurisdictions without a SEP [16].

In order to better characterize the recent increases in HCV infection among young people in California, we surveyed newly reported HCV cases aiming to describe their demographics, risk behaviors, and harm reduction services awareness, access, and utilization to support targeted HCV prevention and treatment strategies.

## Methods

Local health jurisdictions reported HCV cases to the California Reportable Disease Information Exchange (CalREDIE), a web-based, confidential disease reporting and surveillance system, using the 2016 Council of State and Territorial Epidemiologists surveillance case definition [17, 18]. From June 1–December 31, 2018, California Department of Public Health (CDPH) staff used surveillance data to identify newly reported HCV cases 15–29 years of age. CDPH maintains a chronic hepatitis C registry containing hepatitis C cases reported in California by healthcare providers since 1994 and by laboratories since 2007; records are merged and deduplicated using probabilistic matching with SAS software (SAS Institute Inc., Cary, NC). CDPH defined a case of “newly reported” hepatitis C as a person who was reported to CDPH for the first time and who met the Council of State and Territorial Epidemiologists surveillance case definition for current hepatitis C at the time of first report [19]. LHJs were invited to participate in this enhanced surveillance if they met any of the following criteria indicating heightened population risk for HCV infection: 1) the LHJ’s rate of newly reported HCV infections among persons ages 15–29 was higher than the state average among this age group in 2015; or 2) the county was identified by CDC as “vulnerable” to an HCV or HIV outbreak among PWID [20]; or 3) the county had a rate of heroin-related emergency department visits among people aged 15–29 greater than the state average rate among this age group from 2010 to 2015. LHJ staff called patients by phone the month following the report date to complete a structured questionnaire assessing demographics, HCV risk and clinical history, and preventive services awareness, access, and utilization. Race and ethnicity were self-reported in two separate questions. For the purposes of our analysis, we classified “non-Hispanic White” respondents as individuals who self-reported their race as White and who self-reported their ethnicity as non-Hispanic/Latinx. We classified people of color (POC) as respondents who identified their race as African American or Black, Asian, American Indian or Alaska Native, Native Hawaiian or Pacific Islander, mixed race, or other, and/or who self-reported their ethnicity as Hispanic or Latinx. While we recognize the diversity among POC, including differential experiences of systemic racism and individual-level discrimination that may affect lived experiences of hepatitis C risk and access to preventive services and care, small sample sizes precluded conducting statistical analysis stratified among POC groups [21].

Individuals were excluded from participation and subsequent analysis if they resided outside of the LHJ where their case had been reported, had a previously reported HCV infection in CalREDIE, or were incarcerated in a state prison during the follow-up period. Data were entered into Microsoft Access; descriptive statistics were obtained using R version 4.0.1 (R Foundation for Statistical Computing, Vienna, Austria). Statistical analysis was performed in OpenEpi version 3.01 [22]. Chi-square analyses (or Fischer exact, when any cell size was < 5) tested differences in survey responses between PWID and non-PWID as well as POC vs. non-Hispanic White respondents. Bivariate analysis was conducted with chi-square to assess the association of demographic variables (e.g., gender, health insurance status) with race/ethnicity and self-reported PWID status. Statistical significance was set at a *p*-value < 0.05.

This project was determined to be an extension of routine public health surveillance and non-research by the CDPH Committee for the Protection of Human Subjects.

## Results

Interviews were conducted by 8 of 24 LHJs that met project participation criteria (Supplementary Figure 1). A total of 576 individuals were identified, of whom 104 were excluded per the exclusion criteria. Among 472 total HCV cases who met the inclusion criteria, 114 (24%) completed an interview. The median age of respondents was 26 (IQR: 23–28); 48% of respondents were male (*n* = 55) and 52% were female (*n* = 59), of whom 7 were currently pregnant. Eighty-nine percent of respondents (*n* = 99) reported having health insurance, yet only two had taken HCV antiviral medications (Table 1). Twenty-seven percent (*n* = 31) of all respondents had ever been incarcerated for > 24 h; among those individuals, while incarcerated, 29% (*n* = 9) received a tattoo/piercing and 39% (*n* = 12) injected drugs—all 12 of whom (100%) shared injection equipment while incarcerated (Table 2). Incarceration history varied by PWID status: 68% (*n* = 28) of PWID reported ever having been incarcerated for > 24 h compared with 4% (*n* = 3) of non-PWID (*p* < 0.001) (Table 2).

**Table 1:** Demographics and Clinical Management Among Newly Reported Hepatitis C Cases 15–29 Years of Age in Eight Local Health Jurisdictions in California (Imperial, Lake, Monterey, Orange, Placer, Riverside, San Luis Obispo, and Santa Cruz counties), June–December, 2018

	n	(%)
**Age Group**	***N*** **= 114**	
15–18	4	(3.5)
19–22	20	(17.5)
23–26	50	(43.9)
27–29	40	(35.1)
**Race**^b^		
White	77	(67.5)
Other	16	(14.0)
African American/Black	4	(3.5)
Asian	4	(3.5)
American Indian/Alaskan Native	1	(0.9)
Native Hawaiian/Pacific Islander	1	(0.9)
Multiple/Mixed Race	10	(8.8)
Unknown	1	(0.9)
**Ethnicity**^b^		
Not Hispanic/Latinx	59	(50.9)
Hispanic/Latinx	39	(34.2)
Unknown	16	(14.0)
**Gender and Pregnancy**		
Male	55^a^	(48.2)
Female	59^a^	(51.8)
Currently Pregnant	7/60^a^	(11.7)
**Sexual Orientation**		
Heterosexual	99	(86.8)
Bisexual	7	(6.1)
Gay	4	(3.5)
Other	4	(3.5)
**Highest Level of Education Completed**		
Middle school	8	(7.0)
High school	65	(57.0)
College	31	(27.2)
Graduate school	4	(3.5)
Unknown	6	(5.3)
**Employment Status**		
Employed full-time	46	(40.4)
Unemployed	45	(39.5)
Full-time student	6	(5.2)
Employed part-time	11	(9.6)
Other/Unknown	6	(5.2)
**Clinical Management**		
Currently seeing physician for HCV	46	(40.4)
Ever taken HCV antivirals	2	(1.8)
**Indicators of Acute HCV**		
Positive RNA test^c^	43	(37.7)
Elevated liver function tests (LFTs) or jaundice in past year	26	(22.8)
Any symptom of acute HCV in past year	25	(21.9)
Elevated LFTs/jaundice AND any symptom in past year	18	(15.8)
**Insurance Status**	***n*** **= 111**	
Insured	99	(89.2)
Uninsured	12	(10.8)
**Type of Insurance**	***n*** **= 99**	
Private/parent’s/employer insurance	57	(57.6)
Medi-Cal (California Medicaid)	38	(38.4)
Other/Unsure	4	(4.0)

^a^One person who identifies as male indicated their sex at birth as female. No participants self-identified as transgender, genderqueer, nonbinary, nonconforming, or other gender identity

^b^Six individuals who identified their “race/ethnicity” as “White” did not clearly identify their ethnicity as “Non-Hispanic/Latinx.” In the analysis, these individuals were classified as Non-Hispanic White

^c^HCV RNA results were obtained on March 10th, 2019 from the hepatitis C registry maintained by CDPH. However, negative test results are not reported to CDPH, so no further analysis could be performed

**Table 2:** Self-Reported Hepatitis C Risk Factors Among Newly Reported Hepatitis C Cases 15–29 Years of Age in Eight Local Health Jurisdictions in California (Imperial, Lake, Monterey, Orange, Placer, Riverside, San Luis Obispo, and Santa Cruz counties), Stratified by Injection Drug Use Status, June–December, 2018

	PWID		Non-PWID		Total		***p***-value
	n	(%)	n	(%)	N	(%)	
**Responses to open-ended “How do you think you got HCV?”**^**a**^	***n*** **= 41**		***n*** **= 73**		***N*** **= 114**		
Injection drug use	40	(97.6)	0	(0.0)	40	(35.1)	< 0.001
Sexual transmission	4	(9.8)	15	(20.5)	19	(16.7)	0.217
Transfusion/dialysis	0	(0.0)	0	(0.0)	0	(0.0)	–
Nonprofessional tattoo/piercing	2	(4.9)	8	(11.0)	10	(8.8)	0.459
Needlestick/other occupational exposure	0	(0.0)	7	(9.6)	7	(6.1)	0.079
Maternal transmission	0	(0.0)	2	(2.7)	2	(1.8)	0.816
Other household contact	1	(2.4)	5	(6.8)	6	(5.3)	0.590
Other	1	(2.4)	1	(1.4)	2	(1.8)	> 0.999
Don’t know	0	(0)	44	(60.3)	44	(38.6)	< 0.001
**Risk History**							
Prior to diagnosis, thought they were at risk for HCV	31	(75.6)	6	(8.2)	37	(32.5)	< 0.001
Non-professional tattoo or piercing	18	(43.9)	15	(20.5)	33	(28.9)	0.008
Ever exchanged sex for drugs, money, or housing	4	(9.8)	0	(0.0)	4	(3.5)	0.030
Ever been incarcerated > 24 h	28	(68.3)	3	(4.1)	31	(27.2)	< 0.001
Received tattoo or piercing while incarcerated	8 / 28	(28.6)	1 / 3	(33.3)	9 / 31	(29.0)	> 0.999
Injected drugs while incarcerated	12 / 28	(42.9)	–	–	12 / 31	(38.7)	–
Shared injection equipment while incarcerated	12 / 12	(100.0)	–	–	12 / 12	(100.0)	–
Ever used any drug to get high	41	–	20	(27.4)	61	(53.5)	< 0.001
**Among Those Who Ever Used Any Drug to Get High**	***n*** **= 41**		***n*** **= 20**		***n*** **= 61**		
Used drugs in the past year	32	(78.0)	12	(60.0)	44	(72.1)	0.140
Snorted drugs in the past year	25	(61.0)	5	(25.0)	30	(49.2)	0.008
Shared snorting device in past year	21 / 25	(84.0)	3 / 5	(60.0)	24/30	(80.0)	0.509
Smoked drugs (besides tobacco/marijuana) in past year	26	(63.4)	6	(30.0)	31	(52.5)	0.014
Shared smoking device in past year	24 / 26	(92.3)	3 / 6	(50.0)	27/31	(84.4)	0.068
Injected drugs in the past year	34	(82.9)	**–**	–	**–**	–	–
Shared injection equipment in the past year	28	(68.3)	–	–	–	–	–
Knowledge of accessible needle exchange	15	(36.6)	–	–	–	–	–
Ever used a needle exchange to get injection supplies	15	(36.6)	–	–	–	–	–
In county of residence?	13 / 15	(86.7)	–	–	–	–	–
Ever had access to naloxone while injecting	26	(63.4)	–	–	–	–	–
Ever needed naloxone but did not have access	18	(43.9)	–	–	–	–	–
Ever witnessed an overdose	34	(82.9)	–	–	–	–	–
Been prescribed methadone or buprenorphine for >21d	20	(48.8)	–	–	–	–	–

^a^Respondents could select more than one answer to this question

Forty-nine percent of respondents (*n* = 30) reported snorting drugs in the past year, of whom 80% (*n* = 24) shared snorting devices. Among the 20 respondents who ever used any drug but never injected drugs, 25% (*n* = 5) reported snorting drugs in the past year, of whom 60% (*n* = 3) shared snorting devices. Thirty-six percent (*n* = 41) of all respondents reported ever injecting drugs, of whom 68% (*n* = 28) reported sharing injection equipment. The median age of first injection was 19.5 (range: 12–31; IQR: 18–23). Heroin was the most frequently injected drug (*n* = 30), followed by methamphetamine (*n* = 18). Fewer than half of PWID (*n* = 20) had been prescribed methadone or buprenorphine for more than 21 days. Among the 41 respondents who had ever injected drugs, 63% (*n* = 26) ever had access to naloxone during IDU and 44% (*n* = 18) reported they needed naloxone during an overdose but did not have it; 37% of PWID (*n* = 15) knew of an accessible syringe exchange program. Eight PWID (19.5%) lived in a county that had an SEP in 2018, the year of data collection.

One-third of all respondents—76% of PWID (*n* = 31) and 8% of non-PWID (*n* = 6) (*p* < 0.001)—reported thinking they were at risk for HCV prior to their diagnosis. Among non-PWID, 60% (*n* = 44) reported an unknown mode of transmission; 21% (*n* = 15) reported sexual transmission; 11% (*n* = 8) reported a nonprofessional tattoo/piercing; and 10% (*n* = 7) reported an occupational exposure (Table 2). Although 15 non-PWID had received a nonprofessional tattoo or been incarcerated > 24 h, just two of the fifteen (13%) thought they were at risk for HCV prior to diagnosis and five (33%) cited this as the reason they thought they got HCV.

When comparing non-Hispanic White vs. respondents of color, risk perception prior to diagnosis varied: 44% (*n* = 24) of non-Hispanic White respondents thought they were at risk for HCV prior before diagnosis compared to 22% (*n* = 13) of POC (*p* = 0.011). Further, 57% of non-Hispanic White respondents thought their HCV was due to IDU while 15% of respondents of color thought their HCV was from IDU (*p* < 0.001). Risk factors also varied: 57% of non-Hispanic White respondents reported having injected drugs compared to 17% of POC (*p* < 0.001). A larger percentage of non-Hispanic White PWID than PWID of color reported having ever used a SEP (42% vs. 20%) or ever having access to naloxone during IDU (68% vs. 50%), but these differences were not statistically significant (*p* = 0.386 and *p* = 0.313, respectively) (Table 3).

**Table 3:** Self-Reported Hepatitis C Risk Factors Among Newly Reported Hepatitis C Cases 15–29 Years of Age in Eight Local Health Jurisdictions in California (Imperial, Lake, Monterey, Orange, Placer, Riverside, San Luis Obispo, and Santa Cruz counties), Stratified by Self-Reported Race/Ethnicity, June–December, 2018

	Non-Hispanic White		POC		Total		***p***-value
	n	(%)	n	(%)	N	(%)	
**Responses to open-ended “How do you think you got HCV?”**^**a**^	***n*** **= 54**		***n*** **= 59**		***N*** **= 113**^b^		
Injection drug use	31	(57.4)	9	(15.3)	40	(35.4)	< 0.001
Sexual transmission	7	(13.0)	12	(20.3)	19	(16.8)	0.297
Transfusion/dialysis	0	(0.0)	0	(0)	0	(0)	–
Nonprofessional tattoo/piercing	5	(9.3)	4	(6.8)	9	(8.0)	0.887
Needlestick/other occupational exposure	1	(1.9)	6	(10.2)	7	(6.2)	0.144
Maternal transmission	2	(3.7)	0	(0.0)	2	(1.8)	0.452
Other household contact	3	(5.6)	3	(5.1)	6	(5.3)	> 0.999
Other	1	(2.4)	1	(1.4)	2	(1.8)	> 0.999
Don’t know	15	(27.8)	29	(49.2)	44	(32.7)	0.020
**Risk History**							
Prior to diagnosis, thought they were at risk for HCV	24	(44.4)	13	(22.0)	37	(32.7)	0.011
Non-professional tattoo or piercing	16	(29.6)	16	(27.1)	32	(28.3)	0.767
Ever exchanged sex for drugs, money, or housing	3	(5.6)	1	(1.7)	4	(3.5)	0.553
Ever been incarcerated > 24 h	22	(40.7)	9	(15.3)	31	(27.4)	0.002
Received tattoo or piercing while incarcerated	5 / 22	(22.7)	4 / 9	(44.4)	9 / 31	(29.0)	0.434
Injected drugs while incarcerated	9 / 22	(40.9)	3 / 9	(33.3)	12 / 31	(38.7)	> 0.999
Shared injection equipment while incarcerated	9 / 9	(100.0)	3 / 3	(100.0)	12 / 12	(100.0)	–
Ever injected drugs	31	(57.4)	10	(16.9)	41	(36.0)	< 0.001
Ever used any drug to get high	41	(75.9)	20	(33.3)	61	(54.0)	< 0.001
**Among Those Who Ever Used Any Drug to Get High**	***n*** **= 41**		***n*** **= 20**		***n*** **= 61**		
Used drugs in the past year	33	(80.5)	11	(55.0)	44	(72.1)	0.037
Snorted drugs in the past year	23	(56.1)	7	(35.0)	30	(49.2)	0.122
Shared snorting device in past year	17 / 23	(73.9)	7 / 7	(100.0)	24/30	(80.0)	0.340
Smoked drugs (besides tobacco/marijuana) in past year	22	(53.7)	9	(45.0)	31	(50.8)	0.525
Shared smoking device in past year	19 / 22	(86.4)	8 / 9	(88.9)	27/31	(87.1)	> 0.999
**Among Those Who Ever injected drugs**	***n*** **= 31**		***n*** **= 10**		***n*** **= 41**		
Injected drugs in the past year	26	(83.9)	8	(80.0)	34	(82.9)	> 0.99
Shared injection equipment in the past year	21	(67.7)	7	(70.0)	28	(68.3)	> 0.99
Knowledge of accessible needle exchange	12	(38.7)	3	(30.0)	15	(36.6)	0.920
Ever used a needle exchange to get injection supplies	13	(41.9)	2	(20.0)	15	(36.6)	0.386
In county of residence?	11 / 13	(84.6)	2 / 2	(100.0)	13 / 15	(86.7)	> 0.999
Ever had access to naloxone while injecting	21	(67.7)	5	(50.0)	26	(63.4)	0.313
Ever needed naloxone but did not have access	13	(41.9)	5	(50.0)	18	(43.9)	0.655
Ever witnessed an overdose	27	(87.1)	7	(70.0)	34	(82.9)	0.431
Been prescribed methadone or buprenorphine for >21d	16	(51.6)	4	(40.0)	20	(48.8)	0.786

^a^ Respondents could select more than one answer to this question

^b^ One respondent did not provide their race or ethnicity; they were excluded from the denominator of this race/ethnicity table (but are included in the PWID vs. non-PWID table)

In bivariate analysis, there was no statistical association between race/ethnicity and other demographic variables. Respondents reporting injection drug use were significantly more likely to be non-Hispanic White, but there were no statistical differences among PWID by gender, sexual orientation, highest level of education completed, health insurance status, or county of residence (Supplementary Tables 1 and 2). When stratified by PWID status, pre-diagnosis HCV risk awareness was not associated with race/ethnicity (PWID: *p* > 0.999; non-PWID: *p* > 0.999).

## Discussion

In this assessment of newly reported HCV cases among young people age 15–29 in California, we identified racial disparities in risk perception within overall low awareness, access, and utilization of harm reduction services. Although surveillance reports have described the demographics and geographic distribution of newly reported HCV cases in California, this analysis provides insights into risk factors and missed prevention, care, and treatment opportunities among this important age group [23].

More than one-quarter of respondents reported a history of incarceration, with many getting a tattoo/piercing or injecting drugs with shared syringes while incarcerated, demonstrating the overlapping HCV risk factors in this population. Testing in high HCV prevalence settings, such as drug treatment programs, SEPs, prisons, and jails can identify undiagnosed HCV infections among those who may not be engaged in primary care [24]. Correctional institutions provide critical opportunities for conducting HCV prevention, education, testing, and treatment, including strategies to prevent reinfection during incarceration following virologic cure [25].

More than three-quarters (76%) of PWID knew they were at risk for HCV prior to diagnosis. This is consistent with other studies finding HIV and viral hepatitis risk perception among PWID [26]. However, young PWID in this analysis still became infected with hepatitis C despite being aware of their risk, which may be due in part to low self-reported access to and utilization of harm reduction services among PWID. Non-PWID had critically low pre-diagnosis HCV risk perception (8%), despite having other risk factors such as nonprofessional tattoo/piercing or prior incarceration, demonstrating a large gap in risk perception in this group. Hepatitis C transmission related to sharing of tattoo and injection equipment in state prisons has been well documented [27, 28]. CDC recommends HIV and viral hepatitis health education and risk reduction programming in juvenile and adult correctional settings, including through peer health educators, with an emphasis on education regarding injection-related risk [29]. Similar programming regarding non-injection related bloodborne pathogen exposures may also be needed. National clinical guidelines recommend routine opt-out HCV testing and treatment in prisons and jails, along with harm reduction and evidence-based drug treatment to prevent reinfection, and linkage to follow up care upon release [24]. In California, state prisons began screening all newly incarcerated people for HCV infection at intake as of 2016 [30]. California Correctional Health Care Services hepatitis C care guidelines recommend treatment for everyone with hepatitis C with exceptions based on anticipated life expectancy < 12 months (that cannot be remediated by treating HCV) or anticipated release before the evaluation and course of HCV treatment can be completed [31]. The HCV treatment protocol includes screening for substance use disorder and annual HCV RNA rescreening following successful treatment completion to identify potential reinfections [31].

Some non-PWID (15%) and many PWID (61%) reported sharing devices to snort drugs in the past year. Sharing of contaminated drug-sniffing implements has been identified as a possible route of HCV transmission and could explain some of the cases of HCV among our respondents, especially among non-PWID without other identifiable risk factors for HCV infection [32].

Pre-diagnosis risk perception among non-Hispanic White vs. POC respondents varied significantly, which may be explained in part by differences in injection drug use history. We were unable to find comparable research on racial disparities in HCV risk perception, which highlights the importance of these findings. In addition, a larger percentage of non-Hispanic White PWID than PWID of color reported having ever used a SEP (42% vs. 20%) and ever having access to naloxone during IDU (68% vs. 50%) although these differences were not statistically significant. Other research has found lower utilization of harm reduction services among African American/Black and Latino respondents is due in part to concerns about racial profiling, mistrust of police, and stigma [33]. Therefore, potential racial disparities in preventive services access and utilization among young people living with and at risk for hepatitis C in California warrant further exploration with larger sample sizes.

Overdose rates are increasing among young people in California and nationally, yet 44% of respondents reported not having naloxone at an overdose when they needed it [1, 34]. Strategic expansion of SEP and naloxone access for young PWID is critical, since their mortality risk due to overdose is more acute than for HCV. Both CDC and the National Strategy for the Elimination of Hepatitis B and C call for states to expand access to sterile syringes and safe injection equipment and medications for opioid use disorder to reduce the rate of new hepatitis C infections, yet these services remain unavailable in many geographic areas and settings [35, 36].

There is strong evidence supporting the protective effects of maintenance medications for opioid use disorder for reducing HCV incidence among young PWID, which could present a possible incentive among young PWID for adopting MOUD [37]. However, use of MOUD in adolescents is limited, as is reflected in our study: less than half of PWID had ever been prescribed methadone or buprenorphine for more than 21 days [38]. Our findings found higher MOUD uptake than a 2013 analysis of nearly 140,000 episodes of specialty treatment for heroin or opioid use in the Treatment Episode Data Set, which found only 2.4% of adolescents in treatment for heroin received MOUD, as compared to 26.3% of adults [39]. Many drug treatment programs serve older populations that have been using drugs for many years and programs may not be welcoming or appropriate for adolescents and young adults. Youth-friendly harm reduction services may be needed, with input from young people in their design.

Antiviral treatment for HCV is especially low among young people. Although our surveys were conducted only 1 month following the initial HCV case report, only 2 individuals had received antiviral treatment for their HCV at the time of data collection. This is notable as the survey was conducted several years after the advent of direct-acting antivirals for hepatitis C in 2014. Other studies have found low rates of linkage to care among young people with hepatitis C generally and among young PWID. An analysis of linkage to care following HCV screening in an urban emergency department found young age was among several predictors of linkage failure [40]. A retrospective analysis of patients referred to an infectious disease clinic between 2014 and 2018 found lower linkage to care rates among people under 40 years of age [41]. A study among young PWID in San Francisco showed similarly low antiviral initiation rates: only 9.1% of the two-thirds of PWID with diagnosed infection had initiated treatment [42]. Another cohort analysis had similar findings: according to Morris et al., half of young adult PWID in San Francisco with new HCV infection between 2015 and 2018 accepted a referral to HCV care; ultimately only 8% initiated and completed HCV treatment and achieved cure [43]. Identified HCV treatment barriers included fear of medical establishments, competing basic needs, and delaying care until it was necessary [43]. It is unknown how generalizable these findings are to young PWID across California, where the availability of HCV care for PWID may differ. Housing status was not assessed among our survey respondents. Housing instability has also been found to be negatively associated with successful linkage to HCV care, although emerging models of delivering mobile HCV care to people experiencing homelessness yield promise [44].

This analysis has limitations. Our sample is not necessarily representative of all young people in California: only eight of California’s 61 LHJs participated and their response rates varied. Reported IDU history was low despite injection being the known primary risk factor for HCV and 60% of non-PWID reported not knowing how they were infected. This suggests respondents may have been reluctant to answer questions about socially stigmatized behaviors. Housing status was not assessed, which limited examination of unstable housing and homelessness as potential factors influencing low reported hepatitis C treatment uptake. We were unable to analyze whether geography/county SEP presence was a confounding variable on individual SEP knowledge/access due to limited sample sizes in counties with SEPs.

## Conclusions

These data demonstrate low pre-diagnosis HCV risk perception among young non-PWID with other HCV risk factors, high HCV risk awareness among PWID, and low engagement with harm reduction services among young people with newly reported HCV infections in California, with racial/ethnic disparities in HCV risk perception. While history of drug use or incarceration were common, many reported no identifiable risk factor for HCV infection. Expanded HCV education, prevention, testing, linkages to care, and treatment in settings serving PWID and non-PWID at risk for HCV infection, such as schools, juvenile and adult correctional settings, drug treatment programs, and SEPs, as well as increased access to naloxone and other harm reductions services for youth, especially youth of color, should be explored.

## Supplementary Information

**Additional file 1: Table S1.** Bivariate Analysis by Self-Identified PWID Status Among Newly Reported Hepatitis C Cases 15–29 Years of Age in Eight Local Health Jurisdictions in California (Imperial, Lake, Monterey, Orange, Placer, Riverside, San Luis Obispo, and Santa Cruz counties), June–December, 2018*. **Table S2.** Bivariate Analysis by Self-Identified Race/Ethnicity Among Newly Reported Hepatitis C Cases 15–29 Years of Age in Eight Local Health Jurisdictions in California (Imperial, Lake, Monterey, Orange, Placer, Riverside, San Luis Obispo, and Santa Cruz counties), June–December, 2018. **Figure S1.** Criteria for local health jurisdiction and participant inclusion in survey of young people with newly reported hepatitis C in California and response rates.

## Data Availability

The datasets generated and analyzed during the current study are not publicly available as they were collected as part of routine, confidential public health surveillance by the California Department of Public Health but are available from the corresponding author on reasonable request.

## Abbreviations

CalREDIE: California Reportable Disease Information Exchange
CDC: Centers for Disease Control and Prevention
CDPH: California Department of Public Health
HCV: Hepatitis C Virus
IDU: Injection Drug Use
LHJ: Local Health Jurisdiction
MOUD: Medications for Opioid Use Disorder
POC: People of Color
PWID: People Who Inject Drugs
SEP: Syringe Exchange Program
