# Supplementary material for: Disparities in risk perception and low harm reduction services awareness, access, and utilization among young people with newly reported hepatitis C infections in California, 2018
Source: BMC Public Health. 2021 Jul 21;21:1435. doi: 10.1186/s12889-021-11492-3 (PMC8296725; doi:10.1186/s12889-021-11492-3)
Supplement: Supplementary file 1 — Additional file 1: Table S1. Bivariate Analysis by Self-Identified PWID Status Among Newly Reported Hepatitis C Cases 15–29 Years of Age in Eight Local Health Jurisdictions in California (Imperial, Lake, Monterey, Orange, Placer, Riverside, San Luis Obispo, and Santa Cruz counties), June–December, 2018*. Table S2. Bivariate Analysis by Self-Identified Race/Ethnicity Among Newly Reported Hepatitis C Cases 15–29 Years of Age in Eight Local Health Jurisdictions in California (Imperial, Lake, Monterey, Orange, Placer, Riverside, San Luis Obispo, and Santa Cruz counties), June–December, 2018. Figure S1. Criteria for local health jurisdiction and participant inclusion in survey of young people with newly reported hepatitis C in California and response rates. [file 12889_2021_11492_MOESM1_ESM.docx]

| **Supplementary Table 1: Bivariate Analysis by Self-Identified PWID Status Among Newly Reported Hepatitis C Cases 15-29 Years of Age in Eight Local Health Jurisdictions in California (Imperial, Lake, Monterey, Orange, Placer, Riverside, San Luis Obispo, and Santa Cruz counties), June - December, 2018*** | | | | | | |
| --- | --- | --- | --- | --- | --- | --- |
|  | **PWID** | | **Non-PWID** | |  | |
|  | **n** | **(%)** | **n** | **(%)** | **Chi-square** | **p-value** |
| **Gender** | **n=41** |  | **n=73** |  | 0.751 | 0.386 |
| Female | 19 | (46.3) | 40 | (54.8) |  |  |
| Male | 22 | (53.7) | 33 | (45.2) |  |  |
| **Sexual Orientation** | **n=41** |  | **n=73** |  | 0.122 | 0.727 |
| Heterosexual | 35 | (85.4) | 64 | (87.7) |  |  |
| Gay, lesbian, bisexual, or pansexual | 6 | (14.6) | 9 | (12.3) |  |  |
| **Highest Level of Education Completed** | **n=41** |  | **n=68** |  | 4.785 | 0.091 |
| Middle school | 4 | (9.8) | 5 | (7.3) |  |  |
| High school | 29 | (70.7) | 36 | (52.9) |  |  |
| College or graduate school | 8 | (19.5) | 27 | (39.7) |  |  |
| **Insurance Status** | **n=41** |  | **n=70** |  | 2.373 | 0.124 |
| Insured | 39 | (95.1) | 60 | (85.7) |  |  |
| Uninsured | 2 | (4.9) | 10 | (14.3) |  |  |
| **Race/Ethnicity** | **n=41** |  | **n=72** |  | 19.96 | <0.001 |
| Non-Hispanic White | 31 | (75.6) | 23 | (31.5) |  |  |
| POC | 10 | (24.4) | 49 | (67.1) |  |  |

***County of residence was not displayed in the table due to small sample sizes potentially revealing identifiable information; Chi square=7.783, p=0.351.**

| **Supplementary Table 2: Bivariate Analysis by Self-Identified Race/Ethnicity Among Newly Reported Hepatitis C Cases 15-29 Years of Age in Eight Local Health Jurisdictions in California (Imperial, Lake, Monterey, Orange, Placer, Riverside, San Luis Obispo, and Santa Cruz counties), June - December, 2018** | | | | | | |
| --- | --- | --- | --- | --- | --- | --- |
|  | **Non-Hispanic White** | | **POC** | |  | |
|  | **n** | **(%)** | **n** | **(%)** | **Chi-square** | **p-value** |
| **Gender** | **n=54** |  | **n=59** |  | 0.092 | 0.761 |
| Female | 29 | (53.7) | 30 | (50.8) |  |  |
| Male | 25 | (46.3) | 29 | (49.2) |  |  |
| **Sexual Orientation** | **n=54** |  | **n=59** |  | 1.448 | 0.229 |
| Heterosexual | 49 | (90.7) | 49 | (83.1) |  |  |
| Gay, lesbian, bisexual, or pansexual | 5 | (9.3) | 10 | (16.9) |  |  |
| **Highest Level of Education Completed** | **n=52** |  | **n=56** |  | 1.427 | 0.490 |
| Middle school | 5 | (9.6) | 4 | (7.1) |  |  |
| High school | 33 | (63.5) | 31 | (55.4) |  |  |
| College or graduate school | 14 | (26.2) | 21 | (37.5) |  |  |
| **Insurance Status** | **n=53** |  | **n=58** |  | 2.791 | 0.095 |
| Insured | 50 | (94.3) | 49 | (84.5) |  |  |
| Uninsured | 3 | (5.7) | 9 | (15.5) |  |  |

**Supplementary Figure 1. Criteria for local health jurisdiction and participant inclusion in survey of young people with newly reported hepatitis C in California and response rates**

*
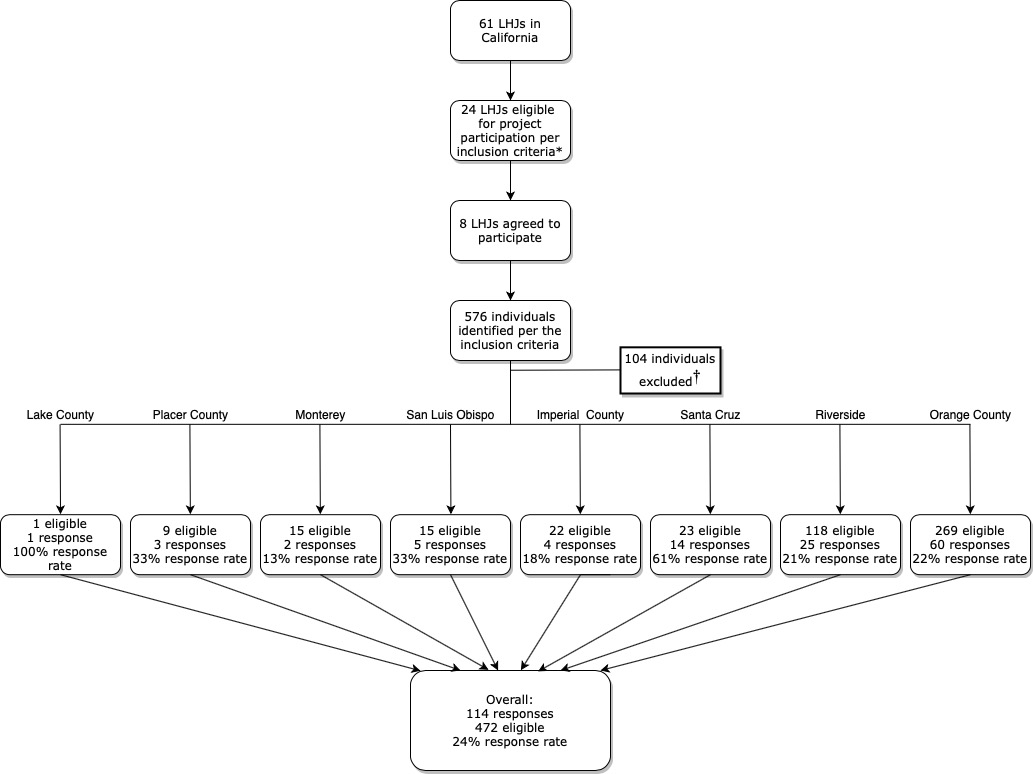
*

***** Inclusion criteria for local health jurisdiction invitation to participate in project:

1. Rate of newly reported HCV infections among persons ages 15-29 higher than the state average among this age group during 2015, or

2. County was identified by CDC as “vulnerable” to an HCV or HIV outbreak among people who inject drugs (PWID), or

3. County had a rate of heroin-related emergency department visits among people aged 15-29 greater than the state average rate among this age group during 2010-2015.

§ Individuals were excluded from follow up if they:

1. Resided outside of the jurisdiction conducting follow-up, or

2. Were incarcerated in a state prison during follow-up, or

3. Had a previously reported HCV infection in California’s chronic hepatitis C disease registry.
